# Clinical efficacy analysis of one-hole split endoscopy surgery versus unilateral biportal endoscopic surgery for degenerative lumbar spondylolisthesis

**DOI:** 10.3389/fsurg.2025.1728502

**Published:** 2026-01-02

**Authors:** Muhaimaiti Abudurezhake, Yifei Huang, Hailong Wang, Gulinuer Aili, Zhanjun Ma

**Affiliations:** 1 The Fourth Clinical Medical College of Xinjiang Medical University, Urumqi, China; 2 Affiliated Hospital of Traditional Chinese Medicine of Xinjiang Medical University, Urumqi, China; 3 Xinjiang Uygur Autonomous Region Institute of Traditional Chinese Medicine, Urumqi, China

**Keywords:** clinical efficacy, lumbar spondylolisthesis, one-hole split endoscopy, retrospective study, unilateral biportal endoscopy

## Abstract

**Objective:**

To compare the clinical efficacy of one-hole split endoscopy (OSE) and unilateral biportal endoscopy (UBE) fusion surgery for degenerative lumbar spondylolisthesis (DLS).

**Methods:**

A retrospective analysis was conducted on 55 patients with DLS admitted between January 2022 and March 2023, including 27 patients in the OSE group and 28 in the UBE group. Perioperative parameters, complications, Visual Analogue Scale (VAS) scores for back and leg pain, Oswestry Disability Index (ODI), and intervertebral space height were recorded and compared preoperatively and at 1 week, 1 month, and 6 months postoperatively. At the final follow-up, clinical outcomes and fusion status were evaluated using the modified MacNab criteria and Bridwell fusion grading system.

**Results:**

No significant differences in baseline characteristics were observed between the two groups (*P* > 0.05). The OSE group demonstrated significantly less intraoperative blood loss (51.25 ± 9.12 mL) and a shorter postoperative hospital stay (3.1 ± 0.8 days) compared to the UBE group (*P* < 0.05). One case of dural tear occurred in the OSE group, while one dural tear and one symptomatic epidural hematoma occurred in the UBE group; all complications resolved with conservative treatment. The mean follow-up duration was 16.0 ± 3.5 months. VAS scores for back and leg pain, ODI, and intervertebral space height showed significant improvement at all postoperative time points compared to preoperative values in both groups (*P* < 0.05). At 1 month postoperatively, the OSE group had a significantly lower VAS score for back pain than the UBE group (*P* < 0.05). No significant intergroup differences were found in other outcome measures at the remaining time points. At the final follow-up, no significant differences were observed in the fusion rate or the excellent-good rate based on the modified MacNab criteria between the two groups.

**Conclusion:**

Both OSE and UBE endoscopic fusion techniques for DLS achieve satisfactory mid- to long-term clinical outcomes and reliable interbody fusion. However, the OSE technique offers minimally invasive advantages, including reduced intraoperative blood loss, faster postoperative recovery, and higher perioperative safety, suggesting it may be a promising alternative for the treatment of DLS.

## 1 Introduction

Degenerative lumbar spondylolisthesis (DLS) is a common cause of low back and leg pain in middle-aged and elderly individuals. Its pathological basis primarily involves the loss of spinal stability resulting from degeneration of the intervertebral discs and facet joints (1). For patients who do not respond to conservative treatment, surgical intervention is an important treatment option. Although traditional posterior lumbar interbody fusion (PLIF) or transforaminal lumbar interbody fusion (TLIF) are effective, they are associated with drawbacks such as extensive muscle dissection, significant intraoperative blood loss, and slow postoperative recovery (2).

With the rapid advancement of minimally invasive spine surgery techniques, endoscope-assisted lumbar interbody fusion has emerged. Unilateral biportal endoscopy (UBE) technique, which establishes two independent channels, enables “endoscopic separation” surgery. It capitalizes on the advantages of both the magnified endoscopic view and the flexible manipulation of conventional instruments, and has been widely adopted for lumbar decompression and fusion surgeries (3, 4). In recent years, One-hole split endoscopy (OSE) has been introduced as a newer minimally invasive technique. It integrates the viewing and working channels into a single sleeve, employing a “single-port, dual-channel” design. This approach theoretically combines the minimal invasiveness of single-portal endoscopy with the procedural convenience of biportal endoscopy (5, 6). Presently, comparative studies focusing on these two mainstream minimally invasive fusion techniques, OSE vs. UBE, for the treatment of DLS are relatively scarce.

Accordingly, we conducted this retrospective cohort study to systematically compare the perioperative outcomes, clinical efficacy, and complication rates between OSE and UBE for endoscopic fusion in treating DLS, aiming to provide evidence for selecting the optimal minimally invasive procedure.

## 2 Materials and methods

### 2.1 General data

A retrospective cohort study was conducted, enrolling patients with single-level DLS who underwent surgical treatment at our hospital between January 2022 and March 2023.

Inclusion criteria: (1) Meeting the diagnostic criteria for DLS with Meyerding grade I or II; (2) Failure to respond to at least 3 months of formal conservative treatment; (3) No associated injuries, no prior spinal surgery, and no severe underlying diseases preoperatively; (4) Complete follow-up data.

Exclusion criteria: (1) Traumatic or pathological spondylolisthesis; (2) History of previous lumbar surgery; (3) Comorbidities such as severe osteoporosis, infection, or tumors.

**Figure 1:**
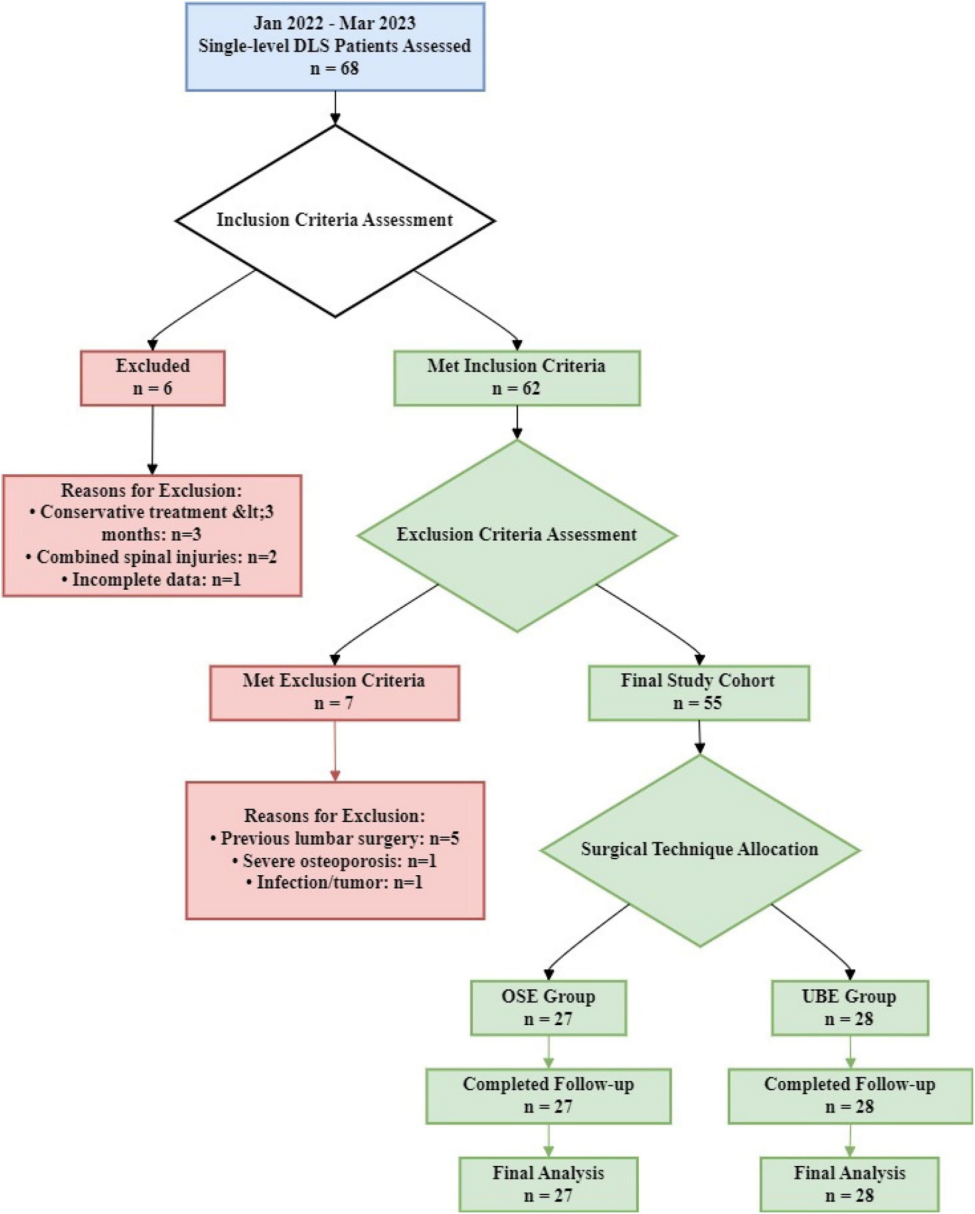
Patient screening flowchart.

A total of 55 patients with lumbar spondylolisthesis were ultimately included in the study. Among them, 27 patients (the OSE group) underwent endoscopic fusion via the OSE technique: 20 males and 7 females, with a BMI of 18.12 ± 2.11 kg/m^2^ and a disease duration ranging from 6 to 14 months. The remaining 28 patients (the UBE group) underwent endoscopic fusion via the UBE technique: 18 males and 10 females, with a BMI of 19.32 ± 2.65 kg/m^2^ and a disease duration of 6 to 15 months. All patients were preoperatively diagnosed with Meyerding Grade I or II lumbar spondylolisthesis. Their clinical presentations included low back pain, lower limb radicular pain, and varying degrees of lower limb sensorimotor deficits. The patient screening process for this study is shown in Figure 1.

The primary basis for assigning patients to either the OSE or UBE technique included: the timing of the surgery (with UBE being more common in the early phase and OSE being increasingly adopted as technical proficiency grew), the anatomical characteristics of the involved segment, the patient's body mass index (BMI), and the surgical team's comprehensive evaluation and preference based on the aforementioned factors.

This study was a retrospective observational study. The study protocol was reviewed and approved by the Xinjiang Uygur Autonomous Region Hospital of Traditional Chinese Medicine. As the research involved only the analysis of pre-existing, de-identified data, did not interfere with patient treatment, and posed minimal risk to patients, it was exempt from formal review by the Ethics Committee. All procedures performed in this study were in accordance with the ethical standards of the Declaration of Helsinki, and stringent measures were taken to protect patient privacy and data security.

### 2.2 Surgical technique

All surgical procedures were performed by the same team of senior attending surgeons, who had each surpassed the learning curve for the respective techniques prior to the commencement of this study, thereby ensuring technical consistency and comparability.Patients in both groups underwent general anesthesia and were placed in a prone position. A Foley catheter was routinely inserted preoperatively.

#### 2.2.1 OSE group

After satisfactory general anesthesia was achieved, the patient was positioned prone. The surgical area was routinely disinfected and draped. The responsible level was precisely localized under C-arm fluoroscopy, and a Kirschner wire was fixed on the lamina as a marker. A longitudinal incision approximately 2.0–2.5 cm in length was made about 1.5–2 cm lateral to the midline on the symptomatic side. Following sequential soft tissue dilation, the OSE portal system was inserted to establish the working channel. The endoscopic imaging system was connected. Under endoscopic visualization, soft tissue over the interlaminar space was meticulously cleared using a plasma radiofrequency probe to adequately expose the superior and inferior laminae and the facet joint. Subsequently, a radiofrequency probe, laminectomy rongeurs, and an endoscopic high-speed drill were utilized to perform a partial laminectomy of the operative level, along with resection of the inferior articular process of the superior vertebra and the superior articular process of the inferior vertebra. Contralateral decompression was performed based on the preoperative plan. The ligamentum flavum was resected, careful hemostasis was maintained, and the dural sac and nerve root were gently exposed and retracted to fully visualize the intervertebral space. The disc space was meticulously prepared using pituitary rongeurs and curettes. The cartilaginous endplates were scraped to create a bleeding bone graft bed, and the harvested autologous bone was morselized for later use. An appropriately sized single cage was selected. The morselized autologous bone combined with allograft bone was packed into the anterior one-third of the intervertebral space, after which the cage was inserted and impacted into place. Satisfactory cage position was confirmed by C-arm fluoroscopy. Exploration confirmed adequate decompression of the dural sac and nerve roots without compression.

Subsequently, pedicle screws were inserted ipsilaterally through the same incision, and percutaneous pedicle screws were placed on the contralateral side. Rods were connected bilaterally, and fluoroscopy again confirmed satisfactory implant position. The surgical field was thoroughly irrigated and checked for hemostasis. A single drainage tube was placed. Finally, the surgical incision was closed in layers.

#### 2.2.2 UBE group

After satisfactory general anesthesia, the patient was placed in a prone position. The surgical area was routinely disinfected and draped. Under C-arm fluoroscopy guidance, the responsible level was precisely identified, and the projections of the superior and inferior pedicles, the intervertebral space, and the midline spinous processes were marked on the skin. Using the intersection of the ipsilateral pedicle line and the intervertebral space as a reference, two transverse skin incisions, each approximately 1 cm long, were made about 1 cm above and below this point. Sequential dilators were then used to bluntly separate the muscle tissue, successfully establishing separate viewing and working portals. A 30° arthroscope was introduced into the viewing portal. A bipolar radiofrequency probe was used for coagulation and to clear soft tissue within the channels, followed by T-handled dissectors to strip the soft tissue from the lamina surface. An osteotome or laminectomy rongeurs were used to resect the partial ipsilateral superior lamina, the inferior articular process, and the medial portion of the superior articular process. The autologous bone harvested during this process was collected for later use as graft material. Contralateral decompression was performed if necessary, based on the preoperative plan. After completing the bony decompression, the ligamentum flavum was removed using Kerrison rongeurs, exposing the dural sac and nerve root. After adequate exposure of the posterior margin of the intervertebral disc, the annulus fibrosus was sharply incised and the nucleus pulposus was removed. Under endoscopic visualization, the cartilaginous endplates were meticulously scraped away until the bony endplates were exposed.Subsequently, bone graft material (National Medical Device Approval No. 20183131638) and harvested autologous bone particles were implanted through the funnel-shaped surgical channel, followed by placement of an interbody fusion cage filled with autologous bone. Satisfactory cage position was confirmed by C-arm fluoroscopy, and exploration verified adequate decompression of the dural sac and nerve roots without compression.

Subsequently, pedicle screws were inserted ipsilaterally through the incision, and percutaneous pedicle screws were placed on the contralateral side. Rods were connected bilaterally, and fluoroscopy confirmed satisfactory implant position. The surgical field was thoroughly checked for hemostasis. A drainage tube was placed. Finally, the surgical incisions were closed in layers.

### 2.3 Outcome measures

Perioperative Parameters: Operative time, intraoperative blood loss, postoperative hospital stay, and the occurrence of complications were recorded.

Efficacy Evaluation: The Visual Analogue Scale (VAS, 0–10) was used to assess the intensity of low back pain and leg pain, and the Oswestry Disability Index (ODI, 0%–100%) was used to evaluate lumbar function. These assessments were conducted preoperatively, at 1 week, 1 month, and 6 months postoperatively. The anterior and posterior heights of the operated intervertebral space were measured on lateral lumbar radiographs preoperatively, at 6 months postoperatively, and at the final follow-up.

Final Follow-up Assessment: Clinical outcomes were evaluated using the modified MacNab criteria, which categorize results as excellent, good, fair, or poor. The excellent-good rate was calculated as (number of excellent + number of good)/total number of cases × 100%. Interbody fusion status was assessed according to the Bridwell fusion grading system (Grade I: fused, Grade II: probably fused), The assessment of interbody fusion in this study primarily relied on lateral lumbar radiographs, following the Bridwell criteria. However, it should be noted that compared to computed tomography (CT), evaluation based on plain radiographs may potentially overestimate the fusion rate, which constitutes a limitation of this study. Future studies utilizing CT assessment will provide more precise evidence regarding fusion status.

### 2.4 Statistical analysis

Statistical analyses were performed using SPSS (Version 26.0). Continuous variables were assessed for normality with the Shapiro–Wilk test. Data conforming to a normal distribution are presented as the mean ± standard deviation and were compared between groups using the independent-samples *t*-test. Categorical data are expressed as frequencies (percentages) and were compared between groups using the Chi-square test or Fisher's exact test, as appropriate. A two-sided *P*-value of <0.05 was considered statistically significant.

## 3 Results

### 3.1 Comparison of baseline characteristics between the two groups

No statistically significant differences were observed between the two groups in terms of gender, age, body mass index (BMI), Meyerding grade, preoperative VAS scores for low back and leg pain, ODI, or intervertebral space height (*P* > 0.05), indicating that the groups were comparable (Table 1).

**Table 1:** Comparison of baseline data between the two groups.

Characteristic	OSE group (*n* = 27)	UBE group (*n* = 28)	Statistic	*P-value*
Gender (Male/Female, *n*)	20/7	18/10	*χ*^2^ = 2.12	>0.05
Age (Mean ± SD, years)	53.43 ± 4.23	55.22 ± 5.11	*t* = −0.44	>0.05
BMI (Mean ± SD, kg/m^2^)	18.12 ± 2.11	19.32 ± 2.65	*t* = −1.22	>0.05
Meyerding Grade (Grade I/II, *n*)	17/10	19/9	χ^2^ = 0.201	>0.05
Lower Limb Symptoms (Unilateral/Bilateral, *n*)	13/14	13/15	χ^2^ = 1.98	>0.05
Decompression Side (Unilateral/Bilateral, *n*)	10/17	13/15	χ^2^ = 1.12	>0.05
Preoperative Low Back Pain VAS Score (Mean ± SD)	5.54 ± 0.98	5.62 ± 0.86	*t* = −0.75	>0.05
Preoperative Leg Pain VAS Score (Mean ± SD)	5.22 ± 0.76	5.76 ± 0.75	*t* = −0.78	>0.05
Preoperative ODI (Mean ± SD, %)	38.65 ± 4.23	37.66 ± 6.12	*t* = 0.49	>0.05
Preoperative Anterior Disc Height (Mean ± SD, mm)	7.44 ± 0.68	7.92 ± 0.88	*t* = −0.87	>0.05
Preoperative Posterior Disc Height (Mean ± SD, mm)	4.62 ± 0.32	4.97 ± 0.37	*t* = −0.54	>0.05

### 3.2 Comparison of perioperative outcomes

The OSE group demonstrated significantly better outcomes than the UBE group in terms of intraoperative blood loss and postoperative hospital stay, with statistically significant differences (*P* < 0.05). No statistically significant difference was found in operative time between the two groups (*P* > 0.05) (See Table 2).

**Table 2:** Comparison of perioperative indicators between the two groups.

Outcome measures	OSE Group (*n* = 27)	UBE Group (*n* = 28)	*P*-value
Operative time (min)	117.83 ± 8.65	121.65 ± 8.59	0.106
Intraoperative blood loss (mL)	51.25 ± 9.12	58.65 ± 6.33	0.001
Postoperative hospital stay (days)	3.1 ± 0.8	4.2 ± 1.1	<0.05

### 3.3 Comparison of complications between the two groups

In the OSE group, one case (3.7%) of dural tear occurred intraoperatively. In the UBE group, one case (3.6%) of dural tear and one case (3.6%) of symptomatic epidural hematoma were observed. All complications were successfully managed, either via intraoperative repair or with postoperative conservative treatment, and no patient required secondary surgery. The difference in the overall complication rate between the two groups was not statistically significant (*P* > 0.05). Both groups of patients achieved favorable surgical outcomes, with representative imaging data from typical cases presented in Supplementary Figure S1 (OSE), Supplementary Figure S2 (UBE).

### 3.4 Comparison of efficacy evaluation indicators between the two groups

At all postoperative time points, both groups demonstrated statistically significant improvement compared to preoperative levels in low back pain VAS scores, leg pain VAS scores, and ODI (*P* < 0.05). Intergroup comparison showed that the postoperative low back pain VAS score in the OSE group was significantly lower than that in the UBE group (*P* < 0.05).Both the anterior disc height measured postoperatively and at 6 months postoperatively showed a significant increase compared to preoperative values in both groups (*P* < 0.05), but no statistically significant difference was observed in these measurements between the groups (*P* > 0.05) (See Tables 3–5).

**Table 3:** Comparison of low back pain VAS scores before and after surgery between the two groups (x¯ ± s, points).

Group	Preoperative	1 week postoperative	1 month postoperative	6 months postoperative
OSE group	5.54 ± 0.98	3.45 ± 1.22	3.01 ± 1.65	1.14 ± 1.01
UBE group	5.62 ± 0.86	5.01 ± 1.41	4.24 ± 1.22	2.24 ± 1.09
*P*-value	0.751	0.012	0.021	0.023

**Table 4:** Comparison of ODI scores between the two groups before and after surgery (x¯ ± s, %).

Group	Preoperative	1 week postoperative	1 month postoperative	6 months postoperative
OSE group	38.65 ± 4.23	32.5 ± 4.45	23.01 ± 5.65	15.9 ± 4.01
UBE group	37.66 ± 6.12	33.4 ± 4.27	24.24 ± 5.22	16.2 ± 4.13
*P*-value	0.490	0.332	0.348	0.502

**Table 5:** Comparison of anterior disc space height between the two groups (x¯ ± s, mm).

Anterior disc height	OSE group (*n* = 27)	UBE group (*n* = 28)	*P*-value
Preoperative	7.44 ± 0.68	7.92 ± 0.88	>0.05
Postoperative	12.14 ± 0.34	12.31 ± 0.25	>0.05
6 Months Postoperative	11.21 ± 0.25	11.26 ± 0.0.31	>0.05

### 3.5 Clinical outcomes and fusion status at final follow-up

At the final follow-up, according to the modified MacNab criteria, the excellent-good rate was 92.6% (25/27) in the OSE group and 89.3% (25/28) in the UBE group, with no statistically significant difference between the two groups (*P* > 0.05). All patients achieved bony fusion (Bridwell grade I or II), resulting in a fusion rate of 100% in both groups. The difference in fusion rates between the groups was not statistically significant (*P* > 0.05) (See Tables 6, 7).

**Table 6:** Evaluation according to the modified MacNab criteria in both groups.

Group	Cases (*n*)	Excellent (*n*)	Good (*n*)	Fair (*n*)	Excellent-good rate (%)
OSE group	27	15	10	2	92.5%
UBE group	28	17	9	2	92.8%
*P*-value		0.671	0.851	0.967	0.967

**Table 7:** Interbody fusion rates in both groups.

Outcome measures	OSE group (*n* = 27)	UBE group (*n* = 28)	*P*-value
Interbody Fusion Status (Bridwell Grade I/II, *n*)	18/9	20/8	0.453

## 4 Discussion

This retrospective study compared the outcomes of OSE and UBE techniques for single-level DLS. The results demonstrate that both minimally invasive endoscopic fusion approaches—OSE and UBE—are effective in alleviating pain, improving functional status, maintaining intervertebral space height, and achieving reliable interbody fusion, with comparable mid- to long-term efficacy. Both techniques utilize a fluid medium for endoscopic operation, which provides excellent visualization and enables precise decompression, while simultaneously maximizing the preservation of the posterior ligamentous complex and muscular integrity. This aligns with the core advantages of minimally invasive spine surgery (7).

A key finding of this study lies in the differences observed in perioperative outcomes. The OSE group demonstrated significant superiority over the UBE group in terms of both intraoperative blood loss and postoperative hospital stay. The underlying reasons may be attributed to the following: ① The single-port design of OSE causes less tissue trauma and potentially less disturbance to the muscle and epidural venous plexus compared to the dual-portal manipulation in UBE, consequently minimizing intraoperative oozing (8), Although the OSE group demonstrated a statistically significant reduction in intraoperative blood loss compared to the UBE group, the absolute difference of approximately 7 mL is likely not clinically significant and is unlikely to have a substantial impact on patient perioperative management or recovery; ②In addition, since both groups of patients were subjected to the same discharge criteria (such as drainage volume less than 50 mL/24 h, ability to ambulate independently, and pain adequately controlled with oral analgesics), the shorter postoperative hospital stay in the OSE group compared to the UBE group suggests that this reduction is more likely attributable to an actual faster recovery rate in the OSE group, rather than being influenced by different discharge protocols (9). The superior low-back pain VAS scores in the OSE group can be attributed to its more minimally invasive surgical approach, which induces less postoperative local soft tissue edema and a milder pain response (10, 11).

Regarding complications, only one dural tear occurred in the OSE group, whereas the UBE group experienced one dural tear and one symptomatic epidural hematoma. Although the intergroup difference was not statistically significant—potentially due to the sample size—this trend remains noteworthy. The UBE technique offers a relatively larger working space and more flexible instrument manipulation. However, the process of establishing dual portals, involving muscle stripping and exposure of the interlaminar space, might increase the risk of injury to the epidural venous plexus and subsequent bleeding. Inadequate hemostasis in such cases could lead to postoperative hematoma formation (12). In contrast, while instrument interference might occur within its single portal, the integrated design of OSE causes less disturbance to the surrounding structures, potentially helping to mitigate such risks (13).It must be emphasized that this study did not demonstrate a statistically significant difference in the incidence of complications between the two techniques. However, this is likely attributable to the limited sample size, which resulted in insufficient statistical power. According to a *post hoc* power analysis, the sample size required to detect the observed differences in complication rates (such as symptomatic epidural hematoma) would be substantially larger than the current cohort. Therefore, it cannot be inferred from these results that the two techniques are equivalent in terms of safety. Future randomized controlled trials with larger sample sizes are needed to more conclusively compare the risk of complications between the two techniques.

On one hand, the phenomenon of “instrument interference” presents a notable challenge during the initial learning curve of the OSE technique. As the OSE system integrates both the endoscopic viewing channel and the working channel into a single sleeve approximately 2.5 cm in diameter, all instruments must operate in coordination with the endoscope within this confined space. While this “single-port working mode” offers significant minimally invasive benefits, it simultaneously carries the risk of collisions between instruments and between instruments and the endoscope itself (14). This challenge is particularly pronounced during procedures requiring extensive instrument manipulation, such as contralateral decompression or endplate preparation, where the shafts of the instruments may obstruct the endoscopic view or impede each other's movement. Consequently, surgeons must possess proficient triangulation skills and exceptional hand-eye coordination to master fine bimanual manipulation and avoid mutual interference (15).

On the other hand, the challenge of “portal convergence” in UBE technology reflects a different operational philosophy. The UBE technique often feels familiar to surgeons transitioning from microscopic or other endoscopic techniques, as its operative principles more closely resemble those of open surgery, achieving separation of the visual field and instrument manipulation (16). However, a key hurdle on its learning curve lies in ensuring the precise “convergence” of the two portals—the viewing portal and the working portal—within the deep anatomical target area. Novice surgeons frequently encounter situations where they “can see the target but cannot reach it” or “have the instrument in place but lose the visual field”, often due to inappropriate interportal angles or errors in depth perception. Consequently, this demands that surgeons possess excellent three-dimensional spatial reasoning, enabling them to mentally visualize and precisely plan the trajectory, angle, and depth of each portal before establishment, ensuring the instrument tip can accurately reach the target area under endoscopic guidance (17). Once successful “convergence” is achieved, the subsequent expansive working space and remarkable instrument flexibility become distinct advantages.

Therefore, the choice of technique for surgeons should be based on their training background and experience. Surgeons proficient in the UBE technique, who are accustomed to the “bimanual separated operation” and “spatial triangulation” mode, need to readjust to the new paradigm of “bimanual coordination within a single portal” and overcome the challenge of instrument interference when transitioning to OSE. Conversely, surgeons accustomed to single-portal endoscopy must conquer the hurdle of “establishing and achieving convergence of dual portals” when learning UBE. In this study, all procedures were performed by the same surgical team that had already surpassed the learning curve for each respective technique, thereby ensuring the comparability of the surgical outcomes. This also highlights that institutional mentorship and structured training systems are crucial for the successful adoption and dissemination of any new technique. Regarding percutaneous screw placement, both techniques require image-guided insertion. The extent of facet joint violation is primarily determined by the screw entry point and trajectory design, rather than the specific endoscopic decompression technique itself. Future research should focus on optimizing screw placement strategies across different technical approaches to maximize the preservation of posterior stabilizing structures.

Limitations of this study include: (1) The limited sample size may have underpowered the statistical analysis for outcomes such as complication rates; (2) The retrospective study design is susceptible to potential selection bias; (3) The follow-up duration was intermediate, lacking longer-term efficacy observations; (4) A cost-effectiveness analysis was not performed; (5) The conclusions of this study are based on surgical outcomes achieved by an experienced surgical team. For novice surgeons, both techniques entail a learning curve, and their initial clinical outcomes may differ from those reported in this study. Future validation through large-sample, multicenter, prospective, randomized controlled trials is warranted.

## 5 Conclusion

In conclusion, both One-hole Split Endoscopy (OSE) and Unilateral Biportal Endoscopy (UBE) are effective minimally invasive approaches for endoscopic lumbar interbody fusion in degenerative lumbar spondylolisthesis, demonstrating comparable radiographic and clinical efficacy in terms of mid- to long-term fusion rates and neurological improvement. However, OSE is associated with an enhanced perioperative profile, characterized by reduced intraoperative blood loss, shorter hospital stay, and more rapid alleviation of early postoperative back pain, positioning it as a valuable surgical option with distinct clinical advantages.

## Data Availability

The raw data supporting the conclusions of this article will be made available by the authors, without undue reservation.
