# Supplementary material for: Clinical efficacy analysis of one-hole split endoscopy surgery versus unilateral biportal endoscopic surgery for degenerative lumbar spondylolisthesis
Source: Front Surg. 2026 Jan 2;12:1728502. doi: 10.3389/fsurg.2025.1728502 (PMC12808485; doi:10.3389/fsurg.2025.1728502)
Supplement: Supplementary file 1 [file Supplementaryfile1.docx]

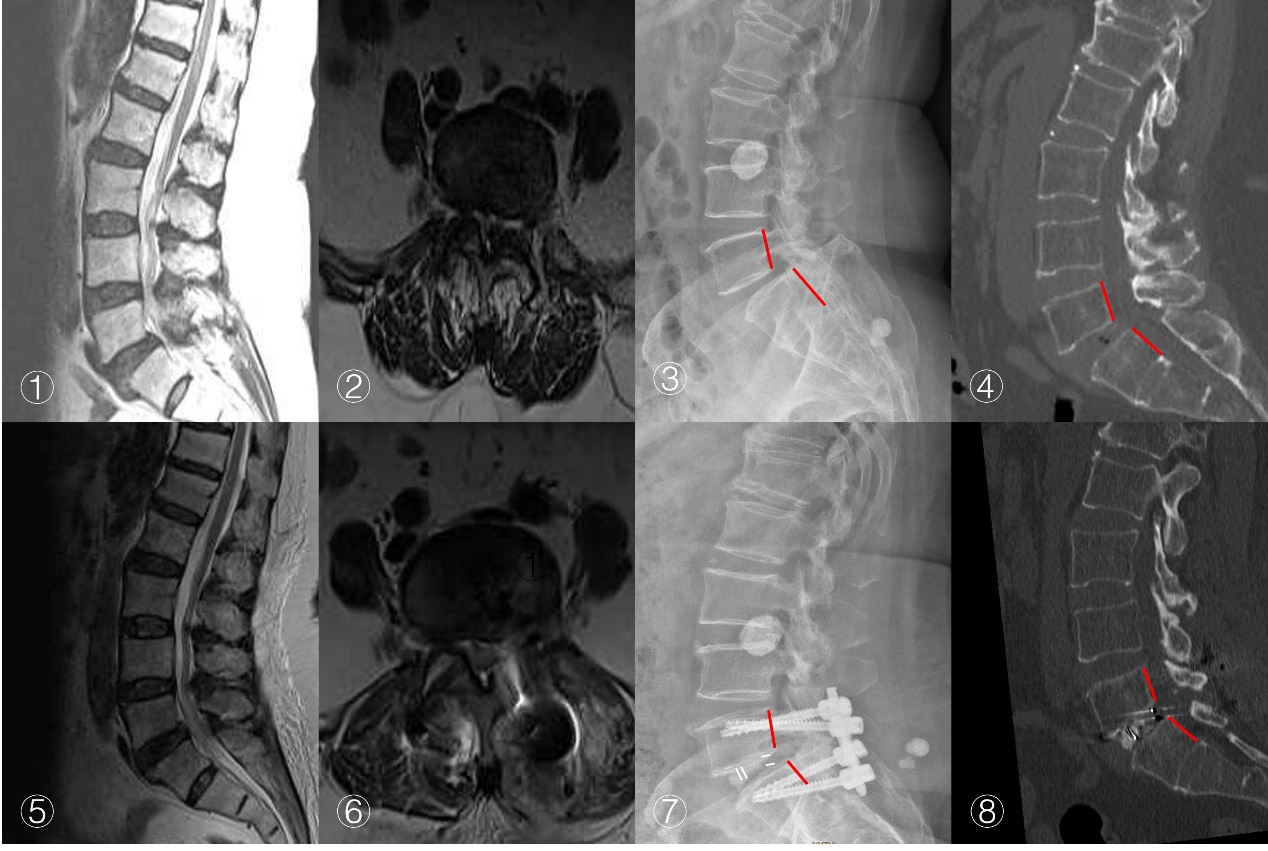


Figure S1（OSE）Representative preoperative and postoperative imaging studies from a patient who underwent One-hole Split Endoscopy (OSE). Panels 1-4 display the preoperative radiographic，CT and magnetic resonance imaging findings. Panels 5-8 demonstrate the corresponding postoperative imaging confirming the final construct.


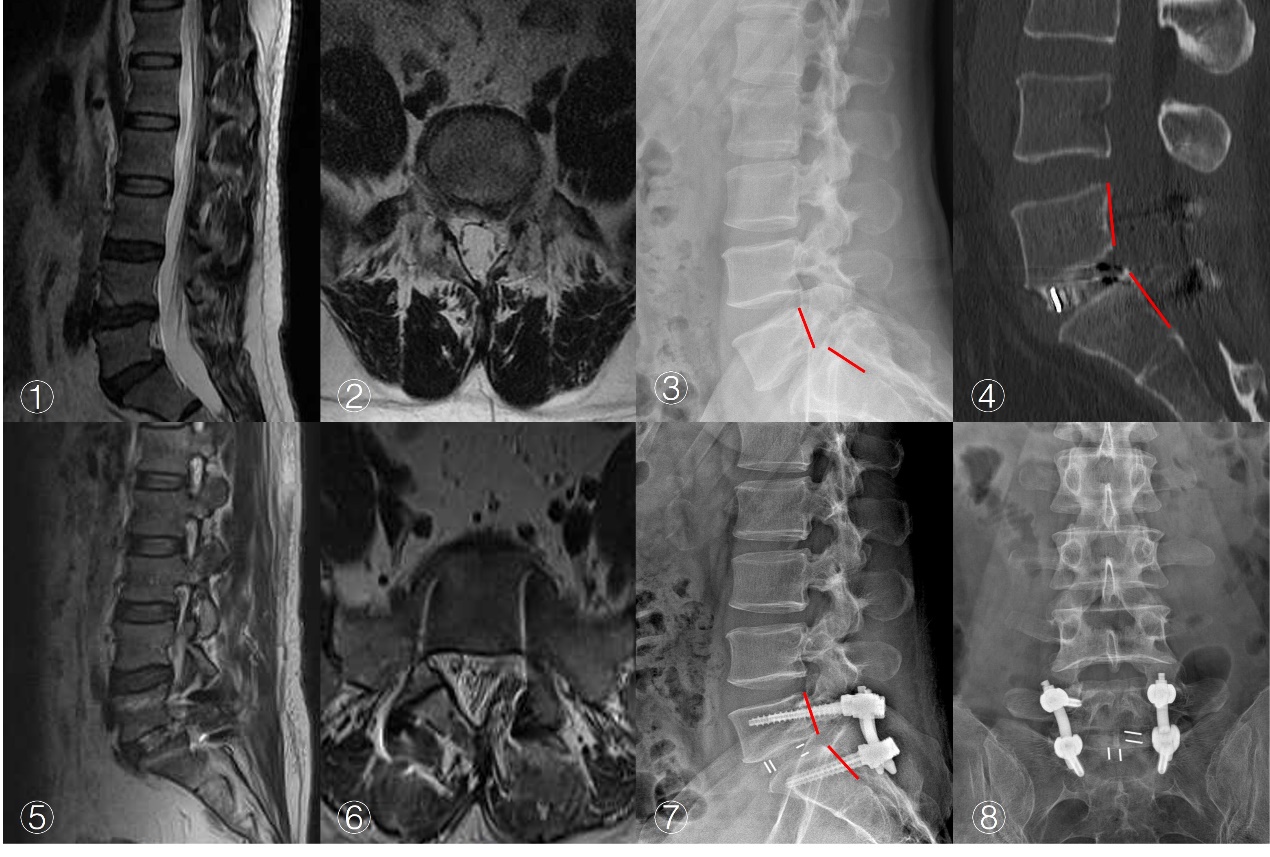


Figure S2（UBE）Representative preoperative and postoperative imaging studies from a patient who underwent Unilateral Biportal Endoscopy (UBE). Panels 1-3 display the preoperative radiographic and magnetic resonance imaging findings. Panels 4-8 demonstrate the corresponding postoperative imaging confirming adequate decompression and final construct.
